# Anaphylaxis management: knowledge of Brazilian pediatricians

**DOI:** 10.1590/1984-0462/2025/43/2024087

**Published:** 2024-12-20

**Authors:** Maria Cecília Barata dos Santos Figueira, Georgia Veras de Araújo Gueiros Lira, Giselia Alves Pontes da Silva, Emídio Cavalcanti de Albuquerque, Luciana Rodrigues Silva, Dirceu Solé, Emanuel Sávio Cavalcanti Sarinho

**Affiliations:** a Instituto de Medicina Integral Professor Fernando Figueira, Recife, PE, Brazil.; b Universidade Federal de Pernambuco, Recife, PE, Brazil.; c Universidade Federal de Bahia, Salvador, BA, Brazil.; d Universidade Federal de São Paulo, São Paulo, SP, Brazil.

**Keywords:** Anaphylaxis, Adrenaline, Knowledge, Management, Pediatricians, Anafilaxia, Adrenalina, Conhecimento, Manejo da doença, Pediatras

## Abstract

**Objective:**

To verify the level of knowledge of Brazilian pediatricians about anaphylaxis, identifying sociodemographic and educational characteristics of the professional which contribute to the adequate management of this clinical disorder.

**Methods:**

A survey was carried out on the management of anaphylaxis using a questionnaire prepared and distributed by email to pediatricians in different states in Brazil. The level of knowledge about anaphylaxis was classified as: satisfactory; unsatisfactory; more than satisfactory; ideal, according to evaluation criteria adopted for the statements of clinical cases that addressed the drug of choice, route of administration, positioning of the patient with anaphylaxis and recognition of the clinical case with differential diagnosis.

**Results:**

A total of 1,645 Brazilian pediatricians participated, of which 48.5% had satisfactory knowledge of the management of anaphylaxis. Approaches to choosing the preferred drug for anaphylaxis and the route of administration were the biggest obstacles to responses. The factors associated with satisfactory knowledge were shorter time since graduation, having a medical residency in pediatrics, having a qualification in allergy and immunology, having completed the Pediatric Advanced Life Support (PALS) in the last two years and having experience in anaphylaxis.

**Conclusions:**

The results regarding satisfactory knowledge of anaphylaxis are lower than those expected for the treatment of a potentially fatal condition. It is important to encourage the continuing education of pediatricians who work mainly in emergencies, wards, and intensive care units, in light of the use of the assumptions of andragogy and Ausubel’s theory, with active methodologies, to promote significant and permanent learning of management of anaphylaxis.

## RESUMO

**Objetivo:**

Verificar o nível de conhecimento dos pediatras brasileiros sobre anafilaxia, identificando características sociodemográficas e educacionais do profissional que contribuem para o adequado manejo desta desordem clínica.

**Métodos:**

Foi realizado um inquérito sobre o manejo da anafilaxia utilizando um questionário elaborado e distribuído por *e-mail* para os pediatras dos diversos estados do Brasil. O nível de conhecimento sobre anafilaxia foi classificado em: satisfatório, não satisfatório, mais que satisfatório e ideal, de acordo com critérios de avaliação adotados para as assertivas dos casos clínicos que abordavam a droga de escolha, via de administração, posicionamento do paciente com anafilaxia e reconhecimento do caso clínico com diagnóstico diferencial.

**Resultados:**

Participaram 1.645 pediatras brasileiros, dos quais 48,5% apresentaram conhecimento satisfatório no manejo da anafilaxia. Abordagens sobre a escolha da droga preferencial na anafilaxia e a via de administração foram os maiores entraves nas respostas. Os fatores associados ao conhecimento satisfatório foram: menor tempo desde a graduação, possuir residência médica em pediatria, possuir habilitação em alergia e imunologia, ter realizado o *Pediatric Advanced Life Support* (PALS) nos últimos dois anos e possuir experiência em anafilaxia.

**Conclusões:**

Os resultados quanto ao conhecimento satisfatório em anafilaxia são inferiores aos esperados para o tratamento de uma condição potencialmente fatal. É importante incentivar a educação permanente dos pediatras que atuam principalmente em emergências, enfermarias e unidades de tratamentos intensivos, à luz da utilização dos pressupostos da andragogia e da teoria de Ausubel, com metodologias ativas, a fim de promover uma aprendizagem significativa e permanente do manejo da anafilaxia.

## INTRODUCTION

As it is a serious and potentially fatal systemic reaction, anaphylaxis becomes an emergency that needs to be quickly recognized and treated by the healthcare professional. Many triggers have been implicated, such as foods, medications, insect bites, immunotherapy with allergens, and latex, among others, and although the incidence of anaphylaxis is increasing in several countries, we do not yet know the magnitude of the results of guidance and awareness interventions that need to be carried out nor have an estimation of aggregate global burdens.^1^

There are several challenges to epidemiological studies on anaphylaxis, as most of the data obtained are from records of patients who were treated or hospitalized in emergency services and may not exactly reflect the incidence in the general population. It is inferred that the lifetime prevalence of anaphylaxis is somewhere between 1.6 and 5.1%, with an average incidence of 50 to 112 episodes per 100,000 people/year, with approximately 25% of cases occurring in patients under 18 years of age, where the prevalence can reach 761/100,000 children/year.^1,2^

Anaphylaxis can occur through immunological (IgE-dependent or not), non-immunological or idiopathic mechanisms. The IgE-dependent mechanism is the one classically described in anaphylaxis and occurs through the binding of IgE to the high-affinity receptors of mast cells and basophils after contact with the identified allergen.^2,3^ All these mechanisms cause the activation of mast cells and basophils with the release of various mediators, such as histamine, arachidonic acid metabolism products, neutral proteases, as well as chemotactic factors, which lead to smooth muscle contraction, vasodilation and increased capillary permeability, causing urticaria, angioedema, flush, itching, wheezing, rhinorrhea, dyspnea, nausea, vomiting, diarrhea, abdominal pain, syncope and hypotension.^4^

Cutaneous symptoms are present in more than 90% of anaphylaxis cases and are normally associated with respiratory and/or cardiovascular and/or gastrointestinal symptoms. Some patients may progress to hypovolemic and distributive shock, with adrenaline being the drug of choice and recommended to be administered first, as it is the only measure capable of reducing hospitalization and mortality. This is due to its effects on alpha-1 adrenergic receptors (promotes selective vasoconstriction and reduction of airway edema); beta-1 adrenergic receptors (positive inotropic and chronotropic effects); and beta-2 adrenergic receptors (decreases the release of inflammatory mediators and promotes bronchodilation). Other measures contribute to improving anaphylaxis. Concomitant to the administration of adrenaline, the trigger for anaphylaxis must be removed, the patient should be properly positioned, oxygen support should be considered, venous access and fluid resuscitation must be installed, and an inhaled short-acting beta-2 agonist drugs should be used, when necessary.^5^

Although definitions and protocols have been published since 2005 based on the results of the First and Second Symposium on the Definition and Management of Anaphylaxis,^6,7^ the 2020 World Allergy Organization Anaphylaxis Assessment and Management Guideline and its updates,^8^ of the EAACI guidelines: Anaphylaxis (2021 update)^9^ and the publication of the Practical Guide for Update on Anaphylaxis by the Brazilian Society of Pediatrics of 2021, anaphylaxis is still underdiagnosed and inadequately treated by doctors.^10^

In surveys of healthcare professionals about the treatment of anaphylaxis, Grossman *et al*. and Derinoz *et al*. found that the percentage of pediatricians who would use adrenaline for simulated clinical cases of anaphylaxis ranged from 24.4 to 93.6%, and the percentage of those who would administer adrenaline intramuscularly corresponds to 43.3–66.9%.^11,12^ In Brazil, a study carried out with pediatricians working in Intensive Care Unit (ICU) found that 83.7% would use adrenaline as a first-line drug in the treatment of anaphylaxis, but only 42% have administered it intramuscularly.^13^

Knowledge about the satisfactory management of anaphylaxis with the use of intramuscular (IM) adrenaline is not uniform among pediatricians. In this sense, the present study proposed to evaluate whether there is a relationship between satisfactory knowledge in the management of anaphylaxis, also considering the patient’s positioning and possible differential diagnoses, and the sociodemographic and professional education characteristics of pediatricians in Brazil.

## METHOD

A survey about the management of anaphylaxis was carried out with pediatricians, using a questionnaire distributed by email to those associated with the Brazilian Society of Pediatrics (SBP) in the different states of Brazil. Information was obtained through a questionnaire sent over a period of three months (between June and August), online, containing multiple choice questions, based on the Anaphylaxis Management of the World Allergy Organization of 2020 and its updates^8^ and the Practical Guide for Update on Anaphylaxis by the Brazilian Society of Pediatrics of 2021^10^ related to anaphylaxis.

Initially, the estimated sample was 380 participants, as there was no previous data on the knowledge of Brazilian pediatricians regarding the treatment of anaphylaxis. It was then subdivided proportionally, according to the size of the extracts (regions of the country). This sample was increased by 10% so that any losses would not compromise its representativeness, totaling 418 individuals. A correction factor of 2.5 (design effect) was also used, as it was a cluster sample, totaling 1,645 pediatricians. For data analysis, all pediatricians who responded to the questionnaire during the study period were included (1,645 pediatricians), exceeding 50% of the estimated sample.

The study was approved by the Human Research Ethics Committee of the Federal University of Pernambuco (Certificate of Presentation for Ethical Appreciation — CAAE: 82218018.7.0000.5208) and followed the recommendations of Resolution 466/12 of the National Health Council.

The instrument applied in the study was developed by the researchers, as there was no validated questionnaire that assessed pediatricians’ knowledge of the topic.

The questionnaire used objective and appropriate language to the study population and objective questions to facilitate data analysis in an optimized time. The first version of the questionnaire was evaluated by two specialists in pediatric allergy and immunology, with adjustments being suggested regarding semantics, sample characterization questions, the content of clinical cases and their response options. After adjustments, the second version was evaluated by a panel composed of ten pediatric allergists and immunologists, members of the Department of Pediatric Allergy of the SBP, who gave their opinion on the suitability of the instrument for assessing knowledge on anaphylaxis. The final instrument was unanimously approved by a panel of experts (content validation). For each question, five equally plausible multiple-choice answer options were provided (the survey can be found in the Supplementary material).

The questions were organized into:

a) characterization of the research subjects and
b) assessment of knowledge in the management of anaphylaxis.

Characterization was divided into sociodemographic and professional education of the participants. Participants were guaranteed anonymity and confidentiality of their responses.

To measure knowledge in the management of anaphylaxis, three clinical cases were presented. The choice of clinical cases aimed to simulate what occurs in clinical practice and used a problem-based approach. Using more than one question on the same topic in a questionnaire reduces the possibility of the participant getting the answers right by chance; therefore, two clinical cases of anaphylaxis (clinical cases 1 and 3) and one clinical case that was not anaphylaxis (clinical case 2) were used to indirectly evaluate the differential diagnosis of anaphylaxis and whether participants would use adrenaline in a patient who was not in anaphylaxis. In each of the scenarios, questions were asked about what the first drug to be administered should be, the route of administration of that drug and the position that should be adopted by the patient.

The level of knowledge was assessed by the answers given to the three clinical cases regarding the drug of choice and the route of administration in clinical cases 1 and 3 of anaphylaxis and classified as:

a) satisfactory knowledge: the participant correctly chose the drug of choice and the route of administration in the two clinical cases of anaphylaxis;
b) unsatisfactory knowledge: the participant answered at least one of the previously mentioned items wrongly;
c) more than satisfactory knowledge: the participant answered correctly the drug of choice, the route of administration and the patient’s positioning in the two clinical cases of anaphylaxis;
d) ideal knowledge: the participant answered correctly the drug, the route of administration and the patient’s positioning in the two clinical cases of anaphylaxis, and recognized clinical case 2 as urticaria with angioedema, with no indication for the use of adrenaline.

The invitation, the informed consent form (TCLE) and the questionnaire were sent by email by SBP, with a link that gave access to the questionnaire on SurveyMonkey to all its members. Biweekly reminders were sent until the end date of the study. The questionnaire was also made available on the SBP website and social media.

At the end of the collection, the data were exported from the SurveyMonkey application to Microsoft Office Excel, version 2010, and later to the STATA/SE 12.0 software. Missing responses were not considered in the analysis.

The results were presented in absolute and relative frequencies. For categorical variables, the Chi-Square or Fisher’s Exact test was used to verify the existence of an association, setting the level of rejection of the null hypothesis at 5%. Taking unsatisfactory knowledge as a parameter, multivariate analysis was carried out using forward logistic regression, including variables with p<0.20 in the analysis.

## RESULTS

The responses to the online questionnaire made it possible to identify the percentage of pediatricians who participated in the research. According to the regions of Brazil, there were 64/1,645 (3.9%) pediatricians in the North region; 271/1,645 (16.5%) in the Northeast region; 158/1,645 (9.6%) in the Central West region; 829/1,645 (50.4%) in the Southeast region and 323/1,645 (19.6%) in the South region.

The median age of Brazilian pediatricians was 42.7 years, ranging from 23 to 78 years, and 78.9% (1298/1,645) were female. The time since graduation ranged from 0 to 54 years, with a median of 17.9 years. The sociodemographic characteristics of professional education are shown in Table 1.

**Table 1:** Sociodemographic and professional education characteristics of pediatricians participating in the study.

Variables	n	%
Time since graduation (years)		
Up to 5	281	17.0
6 to 10	358	21.7
11 to 20	379	23.0
More than 20	627	38.1
Sector in which you work		
Emergency room, infirmary and/or ICU	1158	69.2
Outpatient or office only	352	21.0
I just teach	17	1.0
Health management only	7	0.4
Area in which you work		
Urban	1558	95.3
Rural	16	1.0
Both	61	3.7
Residency in pediatrics	1463	88.5
Qualification in area of activity	970	58.6
Qualification in allergy and immunology	134	8.0
Workplace Medical Residency Program	966	59.3
PALS the last two years	615	37.7
Experience in anaphylaxis	1224	74.7
Brazilian regions		
North	36	59.0
Northeast	116	45.3
Midwest	80	53.3
Southeast	380	48.3
South	148	49.2

ICU:Intensive Care Unit PALS: Pediatric Advanced Life Support.

Table 2 shows the percentage of pediatricians who responded regarding the use of medications necessary for clinical cases of anaphylaxis. Evaluating only the choice of drug, without considering the route of administration, 63.3% responded that they would use adrenaline in the two clinical cases of anaphylaxis.

**Table 2:** Drugs and routes of administration chosen by pediatricians when faced with clinical cases of anaphylaxis.

Drugs and routes	Case 1*		Case 3†	
	n=1595	%	n=1532	%
Adrenaline	1311	79.7	1200	72.9
Intramuscular	1006	76.7	887	73.9
Subcutaneous	260	19.8	193	16.1
Intravenous	26	1.9	117	9.8
Inhalation	19	1.4	3	0.3
Corticosteroid	118	7.4	135	8.7
Beta-2 agonist	90	5.6	99	6.5
Antihistamine	71	4.5	45	2.9
Ranitidine	0	0.0	51	3.3

*In case 1, the number of participants who responded about the drug of choice was 1595, and about the route of administration of adrenaline, 1311;

†In case 3, the number of participants who responded about the drug of choice was 1532, and 1200 responded about the route of administration of adrenaline.

In Table 3, it was possible to identify, in the bivariate analysis, that the time since graduation, having a medical residency in Pediatrics and Allergy/Immunology, in addition to having completed the PALS in the previous two years and having experience in anaphylaxis were statistically significant variables in terms of satisfactory knowledge in management of anaphylaxis.

**Table 3:** Frequency of satisfactory knowledge in the management of anaphylaxis according to the professional education characteristics of pediatricians in the study.

Variables	Satisfactory knowledge		p-value
	n	%	
Time since graduation (years)			
Up to 5	162	60.4	<0.001
6 to 10	214	59.6	
11 to 20	178	48.4	
More than 20	221	36.9	
Area in which you work			
Urban	737	48.6	0.874
Rural	7	43.8	
Both	30	50.8	
Residency in pediatrics	702	49.8	0.012
Qualification in area of activity	455	48.7	0.903
Qualification in allergy and immunology	103	78.6	<0.001
Workplace Medical Residency Program	482	51.0	0.016
PALS the last two years	339	56.2	<0.001
Experience in anaphylaxis	627	52.7	<0.001

PALS: Pediatric Advanced Life Support.

In case 2, in which the patient presented angioedema and urticaria without anaphylaxis, around half of the participants correctly indicated antihistamine in the treatment; however, 35% reported that they would use adrenaline as the first option.

Among the Brazilian pediatricians in the study, 48.5% had satisfactory knowledge of the management of anaphylaxis, 9.5% had unsatisfactory knowledge and 22.2% of participants had more than satisfactory knowledge, agreeing on the drug of choice, the route of administration and the positioning of the patient in the two clinical cases of anaphylaxis. However, when considering ideal knowledge in the management of anaphylaxis, only 19.8% of pediatricians got the three clinical cases right regarding diagnosis, patient positioning, correct therapeutic approach and differential diagnoses.

In Table 4, through multivariate analysis, it can be seen that the time since graduation, and having a qualification in allergy and immunology in addition to experience in anaphylaxis were statistically significant in both groups (more than satisfactory knowledge and satisfactory knowledge). However, having a residency in pediatrics and having completed the PALS in the previous two years were significantly significant among pediatricians who presented more than satisfactory knowledge about the management of anaphylaxis.

**Table 4:** Multivariate analysis of satisfactory and more than satisfactory knowledge according to the professional education characteristics of participants in the study on the approach to anaphylaxis.

Variables*	Knowledge about anaphylaxis			
	More than satisfactory OR (95%CI)	p-value	Satisfactory OR (95%CI)	p-value
Time since graduation				
Up to 5 years	2.59 (1.89–3.56)	<0.001	1.00	<0.001
6 to 10 years	2.31 (1.74–3.05)		1.62 (1.12–2.33)	
11 to 20 years old	1.53 (1.16–2.02)		2.01 (1.51–3.23)	
More than 20 years	1		2.60 (1.84–3.68)	
Residency in pediatrics	1.44 (1.03–2.01)	0.031	-	-
Qualification in allergy and immunology	4.76 (3.00–7.54)	<0.001	4.64 (3.16–6.81)	<0.001
PALS in the last two years	1.32 (1.06–1.64)	0.014	-	-
Experience in anaphylaxis	1.59 (1.25–2.03)	<0.001	1.84 (1.32–2.55)	<0.001
Workplace Medical Residency Program	-	-	1.33 (1.02–1.72)	0.033

OR: odds ratio; 95%CI: 95% confidence interval; PALS: Pediatric Advanced Life Support.

*Variables that started in the model: “time since graduation”, “residency in pediatrics”, “qualification in allergy and immunology”, “PALS in the last two years”, “experience in anaphylaxis”, “workplace residency program” and “works exclusively in outpatient settings”.

## DISCUSSION

Anaphylaxis in the pediatric age group is closely related to the knowledge that every pediatrician needs to have, and investigating the levels of knowledge on the subject allows for educational interventions that can benefit the patient. Considering the assertive use of adrenaline and the route of administration in clinical cases of anaphylaxis, the minimum condition for good medical practice is expected to be satisfactory knowledge of pediatricians in the management of this potentially fatal condition.^14^

According to Ausubel’s learning theory, new information, when incorporated into the set of previous knowledge, enables more meaningful learning. Similarly, the search for best practices in the teaching-learning process based on andragogy enables significant results in knowledge. Therefore, the relevance of a given topic helps with information retention, as it increases motivation to learn.^15^ When it comes to anaphylaxis, it is clear how much this topic needs to be incorporated into the recycling and teaching guidelines in pediatrics.

Less than half of the Brazilian pediatricians who participated in this study (48.5%: 798/1,645) showed satisfactory knowledge in the management of pediatric patients with anaphylaxis. Thisfrequency was higher than the use of IM adrenaline reported in previous studies carried out in Brazil, which ranged from 4 to 41.9%.^13,16,17^ A possible explanation for the improvement in knowledge in recent years must be related to the participation of around 70% of the pediatricians in the study who were working in emergency services, wards and ICUs, and maybe the dissemination by SBP of scientific documents relating to anaphylaxis.^10^

Studies show that, in developed countries such as the United States and Spain, 66.9 and 92.6% of pediatricians, respectively, reported that they would use IM adrenaline as the treatment of choice in anaphylaxis.^11,18^ It is possible that pediatricians in developed countries have greater knowledge about the approach to anaphylaxis due to greater resources, training to work in this condition and availability of adrenaline auto-injectors.

The use of a questionnaire containing three different clinical cases brought a different way of evaluating knowledge about anaphylaxis. Unlike previous studies that used direct questions about the treatment of anaphylaxis, the present study simulated clinical cases, which may have generated doubts in the participants regarding the correct diagnosis of anaphylaxis. Like the patient in case 2, who did not present anaphylaxis, the patient in case 3 did not present cutaneous symptoms, as identified in most cases of anaphylaxis, which may have made his diagnosis difficult for some pediatricians in the study.

The IM use of adrenaline is associated with higher serum levels than the subcutaneous route, and its intravenous use is associated with greater side effects when compared to the IM use. Intravenous adrenaline can be used in patients with hypotension and by experienced professionals in environments with adequate monitoring in ICUs.^5^

Relating the management of anaphylaxis to the characteristics of professional education, our data showed that the shorter the time since graduation, the more likely knowledge will be satisfactory, probably due to younger pediatricians’ access to more recent updates and greater incentives to improve their knowledge and keep updated. In contrast, Olabarri et al. observed a higher rate of success among pediatricians with more than ten years of clinical practice and suggested that younger ones were less experienced with adrenaline outside the ICU environment.^18^

Having a medical residency in Pediatrics and a qualification in Allergy and Immunology were associated with satisfactory knowledge about anaphylaxis, which may be related to academic training in different centers in the country. Such centers follow the recommendations of the National Medical Residency Commission, whose agenda includes knowing how to identify and treat conditions that require advanced life support, such as anaphylactic shock. One explanation for this is the greater possibility of coping with this condition in clinical practice, such as the teaching hospital, which can contribute to significant learning within the context of medical resident training.

Although having taken PALS in the previous two years has been positively associated with satisfactory knowledge about anaphylaxis, a more expressive result was expected, as PALS addresses the treatment of anaphylactic shock, as well as using mixed teaching-learning and simulation methodology (actors and models), which are associated with better knowledge in the long-term management of anaphylaxis.

Among the pediatricians in the study, only 22.2% had more than satisfactory knowledge regarding the patient’s positioning in the management of anaphylaxis, which may be related to the lack of consensus on the Trendelenburg positioning in patients undergoing anaphylaxis. The protocols are controversial regarding the positioning of the patient in anaphylaxis, and some protocols, such as those by Campbell et al.,^19^ do not even mention this aspect of the treatment. The pediatrician’s choice when managing a patient in anaphylaxis who is hemodynamically unstable is to place him in the supine position. In the presence of dyspnea, the position most used by pediatricians is an elevated position, almost sitting, which can often generate confusion in understanding and reflect a lack of pathophysiological knowledge of anaphylaxis.^20^

In terms of limitations of the study, the following were identified: the sample was made up of volunteers associated with the SBP, which did not allow all pediatricians in the country to be included; the absence of a validated questionnaire on pediatricians’ knowledge in the management of anaphylaxis may have contributed to measurement bias. To minimize this bias, the instrument underwent expert evaluation, a strategy that is considered by some authors as content validation. Another limitation refers to information bias, as the questionnaire was available online, which would allow the study participant to carry out research on the topic during responses.

Given the results of the present study, we can conclude that Brazilian pediatricians’ knowledge about the management of anaphylaxis was lower than expected for the treatment of a potentially fatal condition. For participants who presented satisfactory knowledge, the following educational characteristics were listed: shorter time since graduation, residency in pediatrics, qualification in allergy and immunology, having taken the PALS in the last two years and having had previous experience in anaphylaxis.

In recent years, several countries have reported cases of anaphylaxis, which has contributed to more effective intervention measures in public health, such as making auto-injectors available to the population.^21^ In Brazil, in 2021, the Brazilian Association of Allergy and Immunology (ASBAI) created the Brazilian Anaphylaxis Registry, which has made it possible to track the clinical manifestations of the disease in the population, the main etiological agents and the treatments instituted in different age groups, as well as carry out an epidemiological survey of anaphylaxis in Brazil that could raise the awareness of health professionals and managers of this important problem.

It is important to emphasize the need for pediatricians to be up do date, especially those who have worked more than ten years since graduation and who work in hospitals with a pediatric residency program. Similarly, it is necessary to encourage the ongoing education of pediatricians who work in emergency rooms, wards, and ICUs, as well as bring the discussion on the topic to the graduation agenda to stimulate the learning of those in training. Perhaps the use of the assumptions of andragogy and Ausubel’s theory, with active methodologies and within the context of the pediatrician, will promote significant learning and satisfactory knowledge in the management of anaphylaxis. In this sense, there must be an incentive for the continuing education of pediatricians.

## Data Availability

The database that originated the article is available with the corresponding author. CAAE: 82218018.7.0000.5208
